# Adolescent Parenthood and Mental Health in South-Western Uganda: Psychosocial Vulnerabilities, Depression Prevalence, and Associated Factors

**DOI:** 10.7759/cureus.113366

**Published:** 2026-07-25

**Authors:** Scovia Atwiine, Viola N Nyakato, Daniel Atwine, Elizabeth Kemigisha

**Affiliations:** 1 Department of Community Health, Mbarara University of Science and Technology, Mbarara, UGA; 2 Department of Social Work and Social Administration, Bishop Stuart University, Mbarara, UGA; 3 Department of Clinical Research, SRF (Soar Research Foundation) Research and Training Centre, Mbarara, UGA; 4 Department of Health and Wellbeing, African Population and Health Research Center, Nairobi, KEN

**Keywords:** adolescent fathers, adolescent mothers, adolescent parenthood, adolescent parents, adolescent pregnancy, common mental disorders, depression, mental health, psychosocial vulnerability

## Abstract

Background

Adolescent parenthood is associated with adverse psychosocial, economic, and mental health outcomes; however, evidence on adolescent parents remains limited. This study investigated these factors among adolescent parents in South-Western Uganda.

Methods

A cross-sectional study was conducted among 195 adolescent parents, including 155 (79.5%) adolescent mothers and 40 (20.5%) adolescent fathers in South-Western Uganda. The participants were aged 15-19 years. Data were collected using structured interviewer-administered questionnaires. Depressive symptoms were assessed using the Patient Health Questionnaire-9 (PHQ-9) with a score ≥10 indicating depressive symptoms, while common mental disorders (CMD) were assessed using the Shona Symptoms Questionnaire 14 (SSQ-14) with a score ≥8 indicating CMDs. Sociodemographic, socioeconomic, psychosocial, and attitude-related information was also collected. Descriptive statistics were used to summarize participant characteristics. Modified Poisson regression with robust standard errors was used to identify factors independently associated with depressive symptoms.

Results

Participants experienced substantial social and economic vulnerability characterized by school discontinuation, unstable livelihoods, low household assets, food insecurity, and limited support systems. The prevalence of depressive symptoms was 48.2% (n=92), while 72.6% (n=138) screened positive for other common mental disorders. Adolescent mothers had significantly higher depressive symptom prevalence than adolescent fathers (n=79, 52.0% vs n=13, 33.3%; p = 0.048), and severe depressive symptoms were observed only among adolescent mothers. Exposure to violence and mistreatment was common, with many adolescents reporting humiliation, threats, assault, and forced sex/rape. In multivariable analysis, unemployment (adjusted prevalence ratio (aPR) = 2.90, 95% CI: 1.17-7.14), household asset count = 0 (aPR = 2.57, 95% CI: 1.10-6.00), mistreatments by relatives (aPR = 1.52, 95% CI: 1.14-2.02), and mistreatment by neighbours (aPR = 1.43, 95% CI: 1.06-1.95) were independently associated with depressive symptoms.

Conclusion

Adolescent parents in South-Western Uganda experience a high burden of depressive symptoms and common mental disorders within contexts of poverty, violence, stigma, and limited social support. Adolescent mothers appear particularly vulnerable to severe depressive symptoms and forced sex/rape. Comprehensive adolescent-responsive interventions integrating mental health services, economic empowerment, violence prevention, educational reintegration, and community stigma reduction are urgently needed.

## Introduction

Adolescence is a critical stage of human development marked by major biological, psychological, emotional, and social transitions that shape future health and wellbeing. During this period, young people establish personal identity, social relationships, educational goals, and increasing independence while simultaneously adapting to growing societal expectations and responsibilities. However, when adolescence is interrupted by early pregnancy and parenthood, many adolescents face significant psychosocial, educational, economic, and mental health challenges that can negatively affect both their immediate and long-term wellbeing [1]. Across low- and middle-income countries, adolescent pregnancy remains a major public health concern because of its close association with school dropout, poverty, stigma, gender inequality, poor maternal outcomes, and mental health disorders [2].

Globally, depression has become one of the leading causes of illness and disability among adolescents. It contributes to impaired social functioning, poor academic performance, substance use, self-harm, suicidal thoughts, and reduced quality of life [3]. Adolescent parents are particularly vulnerable because they must cope not only with the developmental pressures of adolescence but also with the responsibilities of pregnancy, childbirth, childcare, and economic survival [1]. Evidence from sub-Saharan Africa consistently demonstrates high levels of depression, anxiety, stress, post-traumatic stress symptoms, and suicidal ideation among pregnant and parenting adolescents [4]. Studies from Kenya, Rwanda, Malawi, and Burkina Faso have reported depressive symptom prevalence ranging from approximately 15% to nearly 50%, depending on the context and screening methods used [5].

In Uganda, adolescent pregnancy remains highly prevalent despite national efforts to improve sexual and reproductive health services. Reports continue to show large numbers of adolescents entering parenthood before the age of 19 years, particularly in rural and socioeconomically disadvantaged communities [6]. Ugandan studies further indicate that adolescent mothers commonly experience emotional distress linked to stigma, disrupted education, poverty, intimate partner violence, social exclusion, and inadequate family or community support [7,8]. Adolescent parents in Uganda also face elevated risks of postpartum depression, anxiety disorders, psychological distress, and suicidal thoughts; however, their mental health needs remain poorly integrated within routine maternal and adolescent healthcare services [2].

The mental health burden among adolescent parents is influenced by a complex interaction of individual, family, community, and structural factors. At the individual level, depressive symptoms have been associated with younger age, unintended pregnancy, school dropout, low educational attainment, financial hardship, HIV infection, traumatic experiences, low self-esteem, and substance use [5,9,10] Family-level factors such as lack of parental support, family rejection, partner abandonment, weak family cohesion, and intimate partner violence are also strongly linked to poor mental health outcomes among adolescent parents [2,8,11]. Although adolescent fathers are less frequently studied, available evidence suggests that they also experience substantial psychological distress related to financial responsibility, unemployment, family conflict, and social expectations [12].

Community and societal factors further intensify adolescent vulnerability. Pregnancy-related stigma, community gossip, discrimination from peers and health workers, and social exclusion contribute significantly to emotional suffering and isolation among adolescent parents [8,13]. In many rural African settings, unmarried adolescent motherhood is associated with shame and reduced social acceptance [14]. Negative attitudes from communities may discourage adolescents from seeking healthcare, psychosocial support, or educational reintegration, thereby worsening mental health outcomes, especially for girls [2]. Poverty, unemployment, food insecurity, and unsafe living environments further increase stress and reduce adolescents’ capacity to cope with parenting responsibilities [5, 15].

Despite these challenges, some protective factors have been identified. Supportive family relationships, peer networks, spirituality, community safety nets, and adolescent-friendly services can help reduce depression and psychological distress among adolescent parents [16]. Many adolescents rely on coping strategies such as prayer, resilience, peer support, emotional acceptance, and informal survival mechanisms to manage difficult circumstances [17,18]. However, formal mental health and psychosocial services for adolescent parents remain limited across many African settings, and existing interventions are often fragmented and poorly integrated within maternal and adolescent health programs [2,11].

Importantly, most studies in sub-Saharan Africa have focused mainly on adolescent mothers, with limited attention given to adolescent fathers despite their important psychosocial and economic roles in adolescent parenthood. Additionally, there is limited evidence from South-Western Uganda examining the broader psychosocial vulnerabilities, mental health burden, and social support systems among both adolescent mothers and fathers. This study was therefore conducted to investigate the psychosocial vulnerabilities, mental health burden, and associated factors for depressive symptoms among adolescent parents in South-Western Uganda. The study aimed to generate context-specific evidence that could inform adolescent-responsive mental health programs, psychosocial interventions, and integrated maternal and adolescent healthcare services in Uganda and similar low-resource settings.

## Materials and methods

Study design and setting

Although the overall project used a mixed-methods design, this was a quantitative cross-sectional study conducted in selected health facilities of Bwizibwera Health Centre (HC) IV, Rubindi HCIII, and Rubaya HCIII and surrounding communities in Mbarara District, South-Western Uganda between March 2025 and January 2026. The study was designed to characterize the sociodemographic, socioeconomic, and psychosocial profiles of adolescent parents, determine the prevalence and severity of depressive symptoms and other common mental disorders, and identify factors associated with depressive symptoms among adolescent mothers and fathers in South-Western Uganda. The study population was recruited from the health facility catchment areas and neighbouring communities to include both adolescent mothers and adolescent fathers who were parenting or had been involved in parenting before the age of 19 years.

Study population

The study was designed to include adolescent mothers and fathers aged 10-19 years in South-Western Uganda; however, the study participants were in the age group of 15-19 years, with the majority of participants aged 18-19 years. Adolescents who had become parents before the age of 19 years but were slightly older at the time of data collection were retained because their experiences remained directly related to adolescent pregnancy or parenthood. Participants were eligible if they were adolescent parents, were residents of the study area, and provided informed consent. Adolescents who were slightly older were eligible if they provided informed consent; however, adolescents who were unable to provide informed consent because of severe cognitive or mental impairment were excluded.

Sample Size and Sampling

Participants were recruited through health facilities and community-based structures with support from health workers, Village Health Team members, and local community contacts. Consecutive recruitment was used among eligible adolescents attending selected health facilities, while community mobilization was used to identify adolescent parents who were not actively attending health services. A total of 195 adolescent parents participated in the study, including 155 (79.5%) adolescent mothers and 40 (20.5%) adolescent fathers.

Data collection

Data were collected by trained research assistants using a structured interviewer-administered questionnaire developed by the researchers (see Appendices). The questionnaire collected quantitative data on sociodemographic characteristics, socioeconomic status, schooling, occupation, household assets, deprivation, marital status, family background, sexual and gender-based violence (SGBV) measures, exposure to mistreatment, mental health outcomes, and factors associated with depressive symptoms.

Outcome measures

The primary outcome was depressive symptoms among parenting adolescents. Depressive symptoms were assessed using the Patient Health Questionnaire-9 (PHQ-9), a widely used screening tool for depressive symptom severity. PHQ-9 scores were categorized into minimal, mild, moderate, moderately severe, and severe symptom categories. For prevalence and regression analyses, depressive symptoms were defined as a PHQ-9 score of ≥10 [19]. Other common mental disorders were assessed using the Shona Symptom Questionnaire 14 (SSQ-14). Participants scoring above the established cut-off of ≥8 were classified as screening positive for other common mental disorders [20]. 

Data management and statistical analysis

Data were checked for completeness and consistency before analysis using Stata Statistical Software: Release 15.0 (StataCorp LLC., College Station, Texas, United States). Descriptive statistics were used to summarize participant characteristics. Categorical variables were presented as frequencies and percentages; continuous variables were assessed using histograms. Age was approximately normally distributed and is therefore presented as the mean ± standard deviation.

Participant characteristics were compared between adolescent mothers and adolescent fathers using chi-square tests or Fisher’s exact tests for categorical variables, and differences in median age were assessed using the two-sample Wilcoxon rank-sum (Mann-Whitney U) test. The prevalence of depressive symptoms and other common mental disorders was calculated as proportions with 95% confidence intervals (CIs).

To identify factors associated with depressive symptoms, bivariate analyses were first conducted using chi-square tests and modified Poisson regression with robust standard errors to estimate crude prevalence rates. Variables with statistical or contextual relevance were then included in a multivariable modified Poisson regression model. Adjusted prevalence rates with 95% confidence intervals were reported. Statistical significance was set at p<0.05, and all statistical tests were two-sided

Ethical considerations

The study was approved by the Mbarara University of Science and Technology Research Ethics Committee (approval number: MUST-2024-1387) and the Uganda National Council of Science and Technology (approval number: SS3018ES). Written informed consent was obtained from all the study participants, including the adolescent parents, who were treated as emancipated minors according to section 5.8 of the Uganda National Council for Science and Technology (UNCST) guidelines 2014. Participation was voluntary, and respondents were informed of their right to decline or withdraw without penalty. Privacy and confidentiality were maintained throughout data collection and analysis. Participants with severe depressive symptoms or psychological distress were referred for appropriate counselling and clinical support.

## Results

Participant profile and sociodemographic characteristics

A total of 195 adolescents participated in the study. Most were adolescent mothers (n=155, 79.5%), while 40 (20.5%) were adolescent fathers. Nearly all participants were aged 18-19 years (n=176, 90.3%), although adolescent mothers included a small proportion aged 15-17 years (n=19, 12.3%), while all adolescent fathers were aged 18-19 years (χ²=5.4326, *df*=1, p = 0.015).

Residence differed significantly by participant type. Most adolescent fathers lived in urban areas (n=37, 92.5%) compared with only 38.7% (n=60) of adolescent mothers, while rural residence was reported only among adolescent mothers (n=42, 27.1%) (χ²=37.2215, df=2, p < 0.001). Educational attainment was broadly similar between groups, with most participants having primary education (n=141, 72.3%) (χ²=0.8647, df=2, p= 0.695). Almost all participants were out of school (n=194, 99.5%). Most participants lived in non-owned or non-permanent housing (n=117, 60.0%). Household asset ownership was also limited: 138 (70.8%) participants reported owning only one to two assets, 29 (14.9%) reported at least three assets, and 28 (14.4%) reported no assets, as presented in Table 1.

**Table 1:** Sociodemographic characteristics of participants by participant type. Data given as n (%) unless otherwise marked *p-values based on Fisher’s exact test, **p-value based on two-sample Wilcoxon rank-sum (Mann-Whitney) test. χ²= Chi-square, df= degrees of freedom

Characteristic	Category	Total (n=195), n (%)	Adolescent fathers (n=40), n (%)	Adolescent mothers (n= 155), n (%)	χ²	df	p-value
Age in years Age (years)	mean ± SD	18.6 ± 0.8	18.8 ± 0.4	18.5 ± 0.9	N/A	N/A	0.038
	Median (IQR)	19 (18-19)	19 (19-19)	19 (18-19)	N/A	N/A	0.0863**
Age group (years)	15–17	19 (9.7)	0 (0.0)	19 (12.3)	5.4326	1	0.015*
	18–19	176 (90.3)	40 (100.0)	136 (87.7)			
Area of residence	Urban	97 (49.7)	37 (92.5)	60 (38.7)	37.2215	2	<0.001*
	Peri-urban	56 (28.7)	3 (7.5)	53 (34.2)			
	Rural	42 (21.5)	0 (0.0)	42 (27.1)			
Education level	Never attended school	16 (8.2)	2 (5.0)	14 (9.0)	0.8647	2	0.695*
	Primary	141 (72.3)	29 (72.5)	112 (72.3)			
	Secondary	38 (19.5)	9 (22.5)	29 (18.7)			
Currently in school	No	194 (99.5)	40 (100.0)	154 (99.4)	0.2594	1	1.000*
	Yes	1 (0.5)	0 (0.0)	1 (0.6)			
Occupation	Unemployed	13 (6.7)	3 (7.5)	10 (6.5)	0.3567	2	0.721*
	Self-employed	20 (10.3)	5 (12.5)	15 (9.7)			
	Casual/manual labourer	162 (83.1)	32 (80.0)	130 (83.9)			
Housing status	Not owned/permanent	117 (60.0)	24 (60.0)	93 (60.0)	0.0000	1	1.000
	Owned/permanent	78 (40.0)	16 (40.0)	62 (40.0)			
Household asset count	None	28 (14.4)	5 (12.5)	23 (14.8)	1.0870	2	0.581
	1–2 assets	138 (70.8)	27 (67.5)	111 (71.6)			
	At least 3 assets	29 (14.9)	8 (20.0)	21 (13.6)			

Pregnancy-related experiences and exposure to mistreatment

Areas of Deprivation Within Past Six Months

Table 2 presents the distribution of deprivation experiences among adolescent parents. Overall, deprivation was observed across multiple domains, with notable differences between adolescent fathers and adolescent mothers, particularly in access to healthcare.

**Table 2:** Pregnancy-related experiences and mistreatment characteristics *p-values based on Fisher’s exact test χ²= Chi-square, df=degrees of freedom

Variable	Category	Total (n=195), n (%)	Adolescent fathers (n=40), n (%)	Adolescent mothers (n=155), n (%)	χ²	df	p-value
Deprivation of access to healthcare in last 6 months	No	162 (83.1)	38 (95.0)	124 (80.0)	5.0884	1	0.031*
	Yes	33 (16.9)	2 (5.0)	31 (20.0)			
Deprivation of access to education in last 6 months	No	131 (67.2)	22 (55.0)	109 (70.3)	3.3856	1	0.066
	Yes	64 (32.8)	18 (45.0)	46 (29.7)			
Deprivation of access to food in last 6 months	No	120 (61.5)	29 (72.5)	91 (58.7)	2.5547	1	0.110
	Yes	75 (38.5)	11 (27.5)	64 (41.3)			
Deprivation of access to your child in last 6 months	No	184 (94.4)	37 (92.5)	147 (94.8)	0.3267	1	0.699*
	Yes	11 (5.6)	3 (7.5)	8 (5.2)			
Age in years at first pregnancy/impregnation	10–14	11 (5.6)	1 (2.5)	10 (6.5)	1.0059	2	0.722*
	15–17	149 (76.4)	31 (77.5)	118 (76.1)			
	18–19	35 (18.0)	8 (20.0)	27 (17.4)			
Circumstances in which you became pregnant or impregnated a girl	Willing	118 (61.1)	40 (100.0)	78 (51.0)	32.0705	2	<0.001*
	Tricked/coerced	52 (26.9)	0 (0.0)	52 (34.0)			
	Forced/raped	23 (11.9)	0 (0.0)	23 (15.0)			
Anyone ever done any of the following to you because of your pregnancy or having impregnated a girl	Say or do something to humiliate you in front of others	45 (23.1)	10 (25.0)	35 (22.6)	0.1048	1	0.746
	Threaten to hurt or harm you or someone close to you	72 (36.9)	14 (35.0)	58 (37.4)	0.0799	1	0.777
	Insult you or make you feel bad about yourself	64 (32.8)	16 (40.0)	48 (31.0)	1.1764	1	0.278
	Kick you, drag you, or beat you up	149 (76.4)	37 (92.5)	112 (72.3)	7.2275	1	0.007
	Threatened to attack you with a knife or other weapon	141 (72.3)	33 (82.5)	108 (69.7)	2.6107	1	0.106

Access to Healthcare

Approximately 33 (16.9%) participants reported deprivation in access to healthcare within the past six months. This deprivation was significantly more common among adolescent mothers (n=31, 20.0%) compared to adolescent fathers (n=2, 5.0%), with a statistically significant difference (χ²=5.0884, df=1, p=0.031). Conversely, the vast majority of adolescent fathers (n=38, 95.0%) reported no deprivation compared to 124 (80.0%) adolescent mothers. These findings suggest that adolescent mothers face disproportionately greater barriers to accessing healthcare services.

Access to Education

Nearly one-third of participants (n=64, 32.8%) reported deprivation in access to education. A higher proportion of adolescent fathers (n=18, 45.0%) experienced educational deprivation compared to adolescent mothers (n=46, 29.7%). Although this difference approached statistical significance, it did not reach conventional thresholds (χ²=3.3856, df=1, p=0.066). This pattern indicates a tendency toward greater disruption of education among adolescent fathers, possibly reflecting early entry into income-generating roles or caregiving responsibilities.

Access to Food

Food deprivation was reported by 75 (38.5%) participants, making it one of the most common forms of deprivation. A larger proportion of adolescent mothers (n=64, 41.3%) reported food deprivation compared to adolescent fathers (n=11, 27.5%). However, this difference was not statistically significant (χ²=2.5547, df=1, p = 0.110). Despite the lack of statistical significance, the relatively high prevalence highlights substantial food insecurity among parenting adolescents, particularly mothers.

Access to Their Child

Only a small proportion of participants (n=11, 5.6%) reported deprivation in access to their child. This was slightly higher among adolescent fathers (n=3, 7.5%) compared to adolescent mothers (n=8, 5.2%), though the difference was not statistically significant (χ²=0.3267, df=1, p = 0.699). The high proportion of participants reporting no deprivation (>90% in both groups) suggests that most adolescent parents maintained physical access to their children.

Exposure to Mistreatment

The mean age at first pregnancy or first impregnation was 16.5 years overall, with no statistically significant difference between adolescent fathers and mothers (16.7 vs 16.5 years). Most participants first experienced pregnancy or impregnation at 15-17 years (n=149, 76.4%).

Pregnancy circumstances differed sharply by participant type (χ²=32.0705, df=2, p< 0.001). All adolescent fathers (n=40, 100%) reported that the pregnancy/impregnation was willing, compared with 51.0% (n=78) of adolescent mothers. Among adolescent mothers, 52 (34.0%) reported being tricked or coerced, while 23 (15.0%) reported forced sex or rape.

Mistreatment related to pregnancy or impregnating a girl was common. Overall, 149 (76.4%) reported being kicked, and 141 (72.3%) reported being threatened with attack. Being kicked was significantly more common among adolescent fathers than mothers (n=37, 92.5% vs n=112, 72.3%; χ²=7.2275, df=1, p=0.007).

Prevalence of depressive symptoms and other common mental disorders

Among participants with PHQ-9 data, the prevalence of depressive symptoms was 48.2% (n=92) (95% CI: 41.1%-55.3%). Depressive symptoms were more common among adolescent mothers than adolescent fathers (79, 52.0% vs n=13, 33.3%). The prevalence of other common mental disorders, measured using SSQ-14, was high at 72.6% (n=138) (95% CI: 65.8%-78.5%). CMD prevalence was higher among adolescent mothers (n=113, 75.3%) than adolescent fathers (n=25, 62.5%).

PHQ-9 severity distribution showed that 38 (20.1%) had minimal symptoms, 61 (32.3%) mild symptoms, 34 (18.0%) moderate symptoms, 30 (15.9%) moderately severe symptoms, and 26 (13.8%) had severe symptoms. Severe depressive symptoms were observed only among adolescent mothers (n=26, 17.3%), and PHQ-9 severity distribution differed significantly by participant type as illustrated in Table 3.

**Table 3:** Prevalence and severity of depressive symptoms and common mental disorders. PHQ-9: Patient Health Questionnaire-9; CMD: common mental disorders; SSQ-14: Shona Symptom Questionnaire-14

Mental health outcome	Category	Total, n (%)	Adolescent fathers, n (%)	Adolescent mothers, n (%)
Depressive symptoms, PHQ-9 ≥10	No	99 (51.8)	26 (66.7)	73 (48.0)
	Yes	92 (48.2)	13 (33.3)	79 (52.0)
CMD, SSQ-14 above cut-off ≥8	No	52 (27.4)	15 (37.5)	37 (24.7)
	Yes	138 (72.6)	25 (62.5)	113 (75.3)
PHQ-9 severity category	Minimal	38 (20.1)	10 (25.6)	28 (18.7)
	Mild	61 (32.3)	16 (41.0)	45 (30.0)
	Moderate	34 (18.0)	8 (20.5)	26 (17.3)
	Moderately severe	30 (15.9)	5 (12.8)	25 (16.7)
	Severe	26 (13.8)	0 (0.0)	26 (17.3)

Factors associated with depressive symptoms

Of the 195 participants, 191 were included in the analysis. Four participants were excluded because they had missing PHQ-9 data, which precluded classification of the primary outcome (depressive symptoms). Out of 191 participants, 92 (48.2%) had depressive symptoms (PHQ-9 ≥10). Bivariate associations were examined using chi-square tests and modified Poisson regression with robust standard errors to estimate prevalence rates (PRs) (unadjusted PRs). Variables with statistical and contextual relevance were subsequently included in the final multivariable model.

Association Between Sociodemographic Characteristics and Depressive Symptoms

Depressive symptoms were significantly more common among participants residing in peri-urban areas compared to urban areas (unadjusted PR = 1.40, 95% CI: 1.00-1.95; z=1.97, p=0.049). Education level demonstrated a strong gradient, with participants having primary education or less showing nearly twice the risk of depressive symptoms compared to those with post-secondary education (unadjusted PR = 1.96, z=2.39, p=0.017). Participant type showed a borderline association, with adolescent mothers having higher depressive symptoms than fathers, as illustrated in Table 4.

**Table 4:** Results of bivariate analysis for factors associated with depressive symptoms CI: confidence interval; PR: prevalence rate; N/A: not applicable

Variable	Category	No (n=99), n (%)	Yes (n=92), n (%)	Unadjusted PR (95% CI)	Wald Z- statistic	p-value
Participant type	Adolescent father	26 (66.7)	13 (33.3)	1.00	N/A	NA
	Adolescent mother	73 (48.0)	79 (52.0)	1.56 (0.97–2.50)	1.85	0.064
Area of residence	Urban	56 (59.0)	39 (41.1)	1.00	N/A	NA
	Peri-urban	23 (42.6)	31 (57.4)	1.40 (1.00–1.95)	1.97	0.049
	Rural	20 (47.6)	22 (52.4)	1.28 (0.88–1.86)	1.27	0.205
Age category (years)	15–17	11 (57.9)	8 (42.1)	1.00	N/A	NA
	18–19	88 (51.2)	84 (48.8)	1.16 (0.67–2.01)	0.53	0.597
Age at first pregnancy (years)	10–14	6 (54.6)	5 (45.5)	1.00	N/A	NA
	15–17	79 (53.7)	68 (46.3)	1.02 (0.52–1.99)	0.05	0.959
	18–19	14 (42.4)	19 (57.6)	1.27 (0.62–2.58)	0.65	0.515
Education level	Secondary	24 (64.9)	13 (35.1)	1.00	N/A	NA
	Primary	70 (50.7)	68 (49.3)	1.40 (0.88–2.25)	1.41	0.159
	Never attended School	5 (31.3)	11 (68.8)	1.96 (1.13–3.39)	2.39	0.017
Occupation	Self-employed	16 (80.0)	4 (20.0)	1.00	N/A	NA
	Unemployed	3 (25.0)	9 (75.0)	3.75 (1.47–9.58)	2.76	0.006
	Casual labourer	80 (50.3)	79 (49.7)	2.48 (1.02–6.07)	2.00	0.046
Source of upkeep	Husband/boyfriend	6 (75.0)	2 (25.0)	1.00	N/A	NA
	Parents	6 (66.7)	3 (33.3)	1.33 (0.29–6.09)	0.37	0.710
	Casual labor	71 (50.4)	70 (49.6)	1.99 (0.59–6.69)	1.11	0.268
	Grow crops for sale	5 (27.8)	13 (72.2)	2.89 (0.84–9.96)	1.68	0.093
	Petty trade	9 (69.2)	4 (30.8)	1.23 (0.29–5.27)	0.28	0.780
Deprived of food in the past 6 months	No	72 (61.5)	45 (38.5)	1.00	N/A	NA
	Yes	27 (36.5)	47 (63.5)	1.65 (1.24–2.20)	3.42	0.001
Household assets	≥3	23 (82.1)	5 (17.9)	1.00	N/A	NA
	1–2	68 (49.6)	69 (50.4)	2.82 (1.25–6.36)	2.50	0.013
	None	8 (30.8)	18 (69.2)	3.88 (1.68–8.95)	3.17	0.002
Relative perpetrator	No	84 (56.4)	65 (43.6)	1.00	N/A	NA
	Yes	15 (35.7)	27 (64.3)	1.47 (1.10–1.97)	2.61	0.009
Neighbour perpetrator	No	77 (57.0)	58 (43.0)	1.00	N/A	NA
	Yes	22 (39.3)	34 (60.7)	1.41 (1.06–1.88)	2.36	0.018
Partner’s parents	No	88 (55.4)	71 (44.6)	1.00	N/A	N/A
	Yes	11 (34.4)	21 (65.6)	1.47 (1.08–1.99)	2.47	0.014

Association Between Socioeconomic and Household Factors With Depressive Symptoms

Socioeconomic vulnerability was strongly associated with depressive symptoms. Unemployed participants had nearly four times higher risk compared to self-employed individuals (unadjusted PR = 3.75, p = 0.006). Similarly, lack of household assets demonstrated a clear dose-response relationship, with participants having no assets exhibiting almost fourfold increased risk (PR = 3.88, p = 0.002). Deprivation of food was also significantly associated, with those reporting access having a 65% higher risk of depressive symptoms (unadjusted PR = 1.65, p = 0.001).

Association Between Psychosocial and Interpersonal Factors With Depressive Symptoms

Psychosocial experiences were strongly associated with depressive symptoms. Involvement of close social actors was significantly associated with increased risk. Participants reporting harm from relatives (unadjusted PR = 1.47, p = 0.009), neighbours (unadjusted PR = 1.41, p = 0.018), and partners (unadjusted PR = 1.47, p = 0.014) had a significantly higher likelihood of depressive symptoms.

Multivariable Model of Factors Associated with Depressive Symptoms

In the adjusted model, several factors remained independently associated with depressive symptoms. Adolescents who were unemployed had nearly three times higher risk of depressive symptoms compared to those who were self-employed (aPR = 2.90, 95% CI: 1.17-7.14; z=2.31, p=0.021). Similarly, household economic deprivation demonstrated a strong graded relationship, with those reporting no household assets (aPR = 2.57, z=2.18, p=0.030) and one to two assets (aPR = 2.40, z=2.15, p=0.031) having a significantly higher likelihood of depressive symptoms compared to those with at least three assets.

Interpersonal dynamics also played a critical role. Adolescents who reported harmful involvement by relatives had a 52% higher risk of depressive symptoms (aPR = 1.52, z=2.83, p=0.005), while neighbour involvement increased risk by 43% (aPR=1.43, z=2.32, p=0.020). Although access to food showed a trend toward increased risk (aPR=1.30, z=1.84, p=0.066), this did not reach conventional statistical significance. Likewise, participant type and marital status were not independently associated with depressive symptoms after adjustment, as presented in Table 5.

**Table 5:** Final multivariable model of factors associated with depressive symptoms PR: prevalence rate; CI: confidence interval; N/A: not applicable

Variable	Category	Adjusted PR (95% CI)	Wald Z- statistic	p-value
Occupation	Self-employed	1.00	N/A	N/A
	Unemployed	2.90 (1.17 – 7.14)	2.31	0.021
	Casual/Manual labourer	2.04 (0.88 – 4.71)	1.67	0.094
Deprived food in the last 6 months	No	1.00	N/A	N/A
	Yes	1.30 (0.98 – 1.73)	1.84	0.066
Household asset count	≥3 assets	1.00	N/A	N/A
	1–2 assets	2.40 (1.08 – 5.34)	2.15	0.031
	None	2.57 (1.10 – 6.00)	2.18	0.030
Mistreatment by a relative due to your pregnancy or impregnation	No	1.00	N/A	N/A
	Yes	1.52 (1.14 – 2.02)	2.83	0.005
Mistreatment by the neighbor due to your pregnancy or impregnation	No	1.00	N/A	N/A
	Yes	1.43 (1.06 – 1.95)	2.32	0.020
Participant type	Adolescent father	1.00	N/A	N/A
	Adolescent mother	1.36 (0.87 – 2.13)	1.33	0.182
Marital status	Married/staying with partner	1.00	N/A	N/A
	Not married	1.18 (0.88 – 1.57)	1.09	0.274

## Discussion

This study investigated the mental health burden, associated factors, and psychosocial vulnerabilities among parenting adolescents in South-Western Uganda. The findings demonstrate that adolescent parents experience substantial social and economic deprivation, high exposure to violence and mistreatment, limited support systems, and an exceptionally high burden of depressive symptoms and other common mental disorders. The study further highlights important gendered differences between adolescent mothers and adolescent fathers in experiences of pregnancy, mental health burden, and access to support.

This study found a very high prevalence of depressive symptoms among adolescent parents, with almost half screening positive for depressive symptoms and nearly three-quarters screening positive for other common mental disorders. These findings confirm that adolescent parents constitute an extremely vulnerable mental health population requiring urgent attention.

The prevalence observed in this study is higher than many estimates previously reported in sub-Saharan Africa, including findings from Burkina Faso and Malawi where prevalence ranged between 14.5% and 18.8% among pregnant or parenting adolescents [11]; however, it is comparable to studies conducted in Rwanda and Kenya that documented prevalence ranging from approximately 43% to 58% depending on screening tools and settings [5,21]. The differences across studies may reflect contextual variations in poverty, violence exposure, stigma, access to social support, and availability of mental health services, but they collectively point to a substantial and persistent burden of mental illness among adolescent parents.

The high prevalence of common mental disorders observed in this study further emphasizes that the psychological burden experienced by adolescent parents extends beyond depression alone. Previous African studies have similarly documented anxiety disorders, stress, trauma-related disorders, suicidal ideation, emotional dysregulation, and behavioral difficulties among pregnant and parenting adolescents [4]. The prevalence of depressive symptoms and common mental disorders identified in this study suggests that many adolescents experience multiple psychosocial and emotional difficulties simultaneously.

Particularly concerning was the finding that severe depressive symptoms were observed only among adolescent mothers. Adolescent mothers also reported significantly higher overall depressive symptom prevalence than adolescent fathers. This finding aligns with global and African evidence showing that adolescent girls are disproportionately affected by depression due to gendered vulnerabilities including intimate partner violence, caregiving burdens, school discontinuation, social stigma, reproductive coercion, and economic dependence [22,23]. The findings also support the socio-ecological understanding of adolescent mental health, which recognizes that depressive symptoms emerge from interacting individual, family, interpersonal, community and structural vulnerabilities rather than from isolated personal factors alone.

Another notable finding from this study was the high level of violence, coercion, and mistreatment associated with adolescent pregnancy and parenthood. A substantial proportion of adolescent mothers reported being tricked, coerced, or raped, while many participants reported physical assault, humiliation, threats, and violence linked to pregnancy or impregnating a girl. These findings are consistent with qualitative and quantitative studies from Uganda, Kenya and South Africa showing that adolescent pregnancy frequently occurs within contexts of gender inequality, transactional sex, coercion, violence and limited reproductive autonomy [4,24]. Previous Ugandan studies have similarly documented rejection, abandonment, emotional violence and physical abuse directed toward pregnant adolescents by partners, parents and communities [7,25].

The finding that adolescent fathers reported particularly high levels of assault by partners’ parents highlights the social consequences experienced by adolescent boys involved in pregnancy. Although adolescent mothers often carry the greatest physical and psychosocial burden, adolescent fathers may also experience community hostility, pressure, punishment and social exclusion. This suggests that adolescent fatherhood remains an underexplored dimension of adolescent reproductive health requiring greater research and programming attention.

The relationship between violence exposure and depressive symptoms observed in this study also mirrors previous African evidence linking intimate partner violence, physical abuse, stigma and traumatic experiences with poor mental health among adolescents [26]. Violence likely contributes to depression through chronic stress activation, fear, social isolation, low self-worth, and trauma-related psychological responses.

The multivariable findings demonstrate that depressive symptoms among parenting adolescents were independently associated with unemployment, household poverty, and harmful involvement from relatives and neighbours. The strong association between unemployment and depressive symptoms is consistent with literature demonstrating how financial insecurity, dependence, and inability to provide for children contribute to psychological distress among adolescent parents [2,26]. Adolescents who lack income opportunities may experience feelings of hopelessness, social inadequacy, and chronic stress related to caregiving responsibilities. Similarly, the graded association between low household assets and depressive symptoms highlights the role of structural deprivation in shaping adolescent mental health. Poverty influences mental health both directly through material deprivation and indirectly through food insecurity, poor healthcare access, school discontinuation, and increased exposure to violence and exploitation [1,27].

An especially important finding was the independent effect of harmful involvement by relatives or neighbours. Adolescents exposed to mistreatment from relatives or neighbours because of pregnancy or having impregnated someone had significantly higher odds of reporting depressive symptoms. This reflects the damaging mental health impact of community stigma, rejection, blame and discrimination. Previous African studies have shown that pregnancy-related stigma contributes to social isolation, loss of social support, reduced educational opportunities and emotional distress among adolescent mothers [14]. The persistence of interpersonal and community factors after adjustment supports the importance of ecological approaches to adolescent mental health interventions. Mental health among adolescent parents cannot be addressed solely through clinical treatment; it also requires interventions that strengthen family relationships, reduce stigma, improve community support systems, and address poverty and gender inequality.

The findings reveal that adolescent parents in South-Western Uganda live within highly vulnerable social and economic conditions characterized by disrupted education, unstable housing, low household assets, food insecurity, and precarious livelihoods. Nearly all participants were out of school, while most depended on casual labour and lived in non-permanent housing. These findings align with previous studies from Uganda, Malawi, Kenya and South Africa showing that adolescent pregnancy frequently interrupts schooling and pushes adolescents into cycles of poverty, dependence and social exclusion [2,26]. The observed educational disruption is particularly concerning because education has consistently been identified as a protective factor against depression and psychosocial distress among adolescents. Previous studies in Malawi and Burkina Faso demonstrated that adolescents with secondary education were less likely to experience depressive symptoms compared to those with lower educational attainment [15]. Similarly, Indonesian and Ugandan studies have linked low educational attainment with increased depressive symptoms, poor coping capacity and reduced problem-solving abilities [10,28].

The high prevalence of food deprivation and limited household assets observed in this study further reflects the intersection between adolescent parenthood and structural poverty. Food insecurity and economic hardship are well-recognized determinants of poor mental health outcomes among adolescents and young mothers across sub-Saharan Africa [4]. Adolescents who become parents often lose educational opportunities and economic independence, leaving them highly dependent on unstable social support systems. In this study, the fact that many adolescents reported having no reliable emergency support further illustrates the fragility of their social safety nets.

An important gendered pattern emerged whereby adolescent mothers were significantly more likely to report deprivation in access to healthcare, while adolescent fathers more frequently experienced educational deprivation. This finding suggests that adolescent pregnancy and parenthood may affect male and female adolescents differently. Adolescent mothers may experience barriers related to stigma, caregiving burdens, poverty, and judgmental health systems, while adolescent fathers may experience pressure to abandon school and enter low-paying labour roles to provide financially. Similar gendered disruptions have been described in qualitative studies across East and Southern Africa where adolescent fathers experience social pressure to assume provider roles despite lacking economic stability [22].

Strengths and limitations

This study contributes important evidence on the mental health burden and psychosocial vulnerabilities of parenting adolescents in Uganda by including both adolescent mothers and adolescent fathers, an area that remains understudied in African mental health research. The study also examined a broad range of socioeconomic, interpersonal and community-level factors, allowing a more comprehensive understanding of adolescent parenting experiences.

However, several limitations should be acknowledged. First, the cross-sectional design limits causal inference regarding relationships between psychosocial factors and depressive symptoms. Second, the use of self-reported mental health screening tools may have introduced reporting bias or social desirability bias. Third, the relatively smaller number of adolescent fathers compared to mothers may have reduced statistical power for some comparisons. Also, the use of non-probability/consecutive sampling strategy could have left out a big number of adolescents who did not turn up for the study, hence limiting generalizability. Finally, because the study was conducted in selected communities in South-Western Uganda, generalizability to other settings should be interpreted cautiously.

Implications for policy and practice

The findings underscore the urgent need to integrate adolescent mental health services into maternal, reproductive and adolescent health programs in Uganda. Routine mental health screening using validated tools should be incorporated into antenatal, postnatal and adolescent health services. There is also a need for adolescent-friendly psychosocial support systems that address stigma, violence, economic vulnerability and social exclusion. Community-based interventions involving families, peers, schools, local leaders and health workers may help reduce harmful actions and strengthen social support networks.

Economic strengthening interventions, educational reintegration programs and livelihood support may be particularly important for reducing mental health vulnerability among parenting adolescents. Given the strong influence of relatives and neighbours on mental health outcomes, community stigma reduction and parenting support interventions are equally critical. Programs should also intentionally include adolescent fathers, who remain largely neglected within reproductive and mental health programming despite experiencing important psychosocial vulnerabilities.

## Conclusions

Adolescent parents in South-Western Uganda experience a substantial burden of depressive symptoms and other common mental disorders within a broader context of socioeconomic deprivation, violence, stigma, and limited social support. Adolescent mothers bear a particularly heavy burden characterized by higher depressive symptom prevalence, more severe depressive symptoms, greater healthcare deprivation, and increased exposure to coercive and violent pregnancy experiences. Depressive symptoms were independently associated with unemployment, household poverty, and harmful involvement from relatives and neighbours, emphasizing the importance of social and structural determinants of adolescent mental health

The findings further demonstrate that adolescent pregnancy and parenthood are deeply gendered experiences shaped by interpersonal relationships, community perspectives, and broader social inequalities. Addressing the mental health needs of adolescent parents therefore requires comprehensive, multisectoral and adolescent-friendly approaches that combine mental health care, social protection, violence prevention, educational support, economic empowerment and community stigma reduction. Strengthening these systems is essential not only for improving adolescent wellbeing but also for enhancing long-term family, child, and community outcomes

## Appendices

**Table 6:** Study questionnaire for adolescent parents

Date: |__|__|/ |__|__| /|__|__|__|__|	ID: |__|__|__|

No.	Question	
1.	Participant- Adolescent mother |___| , Adolescent father |___|	2. Area of residence: ________________________________
3.	How old are you now? |__|__| years	4.At what age did you first get pregnant or impregnated a girl |__|__| years
5.	What is your highest level of education? Never attended school Primary Secondary Tertiary (university, vocational training) Other specify ___________	6.Are you currently in school 1= Yes (if yes skip to Q 8) 2= No
7.	If No, indicate main reason for being out of school lack of school fees, uniform or materials got married wanted to earn money lost interest not a good student (e.g failed to get good grades) Got pregnant Other specify _____________________	What is your religion? No religion Catholic Protestant Muslim Seventh Day Adventist Pentecostal If other, specify _________________
9.	What do you do for a living (occupation)? Unemployed Self-employed Student Casual laborer Work as a maid e. If other, specify ______________	10. Where do you get most of the money for upkeep? Provided for by husband/boyfriend Provided for by parents Casual labor (e.g., people’s farms) Grow crops for sale Petty trade Own/work in a shop Work in a saloon If other, specify ___________
11.	Type of house you live in? Temporary (grass thatched roof) Semi-permanent (iron roof, wattle walls) Permanent (iron roof with brick walls) Other If other, specify__________	12. In the past 6 months, have you been deprived (or denied the right to have) of the following Housing 1= Yes 0= No Access to healthcare 1=Yes 0=No Access to education 1=Yes 0=No Access to food 1=Yes 0=No Access to your child 1=Yes 0=No Other, specify ________________
13.	If you said any yes in question 11, explain briefly the circumstance or example of the deprivation _____________________________________________	
14.	Household assets: Does your family own any of the following? 1. Yes 0. No 1. Radio |___| 2. Mobile telephone |___| 3. Television |___| 4. Bicycle |___| 5. Chairs with cushions |___|	15.Compared to other families in your community, how do you assess the economic situation of your household now? Very poor compared to others Moderately poor compared to others Not poor, regular, Richer
16.	If you have a problem or an emergency whom do you reach out to for help? Select the most important person No one Boyfriend/ Husband Mother/ Father Sibling Aunt Uncle Friend Other Relatives Neighbours If other, specify _____________	17.What is your marital status? Married Single, skip to Q 21 Living with partner Divorced Separated Widowed Other If other, specify ______________
18.	How old were you when you first got married/started living with a partner as if married? |__|__| years	19.How old is/was your spouse/partner? |__|__| years
20.	What was the main reason you got married for the first time? Love or companionship Mother encouraged it Father encouraged it Was pregnant Financial security Forced by parents/family/ relatives To settle down/start or raise a family Problems at home Dowry payment/Family compensation Other, specify__________________	21.With whom are you currently living? Parents Relatives Partner (husband) Partner’s family If other, specify _________________
22	Are your parents alive? Yes, both are alive No, mother died No, father died Both parents died	23. If both parents are alive, are they living together Yes, they live together under one roof (both biological parents) Yes, they live together (biological parent and non-biological parent) No, they live separate due to polygamy (has another partner) No, they live separate due to separation/divorce Other, specify___________

24.	Thinking about the circumstances in which you became pregnant or impregnated a girl, which statement best describes your experience? I was willing I was tricked I was forced I was raped I was coerced (received money, food, clothing, gifts) I was expected to do it as part of my job I wanted to have a baby Other, specify_____________	25. Has anyone ever done any of the following things to you because of your pregnancy or having impregnated a girl. a. Say or do something to humiliate you in front of others? 1= Yes 0= No				
		Threaten to hurt or harm you or someone close to you? 1= Yes 0= No				
		Insult you or make you feel bad about yourself? 1= Yes 0= No				
		Kick you, drag you, or beat you up? 1= Yes 0= No				
		Threatened to attack you with a knife or other weapon? 1= Yes 0= No				
		26	If any of the above is answered YES, who did those things to you? (Probe more) ____________________________________________					
27.	Have you ever been tested for HIV? 1=Yes 0= No	28. If no what is the main reason for not testing for HIV Fear of drawing blood I do not know where to go for an HIV test Health facility is far Fear to know my result Was told I was born with HIV Other, specify				
29.	If yes, when did you last test for HIV? less than 3 months ago between 3 to 6 months ago between 6 to 12 months ago about a year ago more than one year ago	30. Would you mind sharing with us results of your HIV status 0= Positive 1= Negative, 2= I do not know my status 3= I know my status but do not wish to share				
31.	If positive, Are you currently on ART? 1= Yes 0= No	32. If yes how long have you been on ART since birth less than 1 year 1 to 2 years 3 or more years				
33.	Do you know the status of your current sexual partner? 0= No 1= Yes negative 2=Yes positive 3= Not applicable (not I a relationship) 4=Don’t know	34. Has anyone in your family or close to you became pregnant or impregnated someone in early age (up to 19 years old) Mother Father Aunt Uncle Sister Cousin Relative Other, specify				
35.	Attitudes of parenting adolescents	Agree a lot (1)	Agree (2)	Neither agree nor disagree (3)	Disagree (4)	Disagree a lot (5)
	It is ok to be pregnant at an early age till 19?					
	Young people who get pregnant at an early age are mistreated in this community					
	Young people who get pregnant should not be allowed to attend regular school like those who never got pregnant					
	Young people who get pregnant at an early age have no choice but to get married to anyone available					
	A mother is blamed in this community if her daughter becomes pregnant at a young age					
	Men who get young girls pregnant should be punished by the law					
	Care of children who are born to young people at an early age is a responsibility of the girl/girl’s family					
	Care of children who are born to young people at an early age is a responsibility of the boyfriend/Boyfriend’s family					
	Young people who get pregnant can be successful in future if supported					
	Health workers in our community treat young people who get pregnant fairly (like older mothers)					

36.	During the course of the past weeks (Shona scale for adolescents)	0=No	1=Yes
	Did you have times in which you were thinking deeply or thinking about many things?		
	Did you find yourself sometimes failing to concentrate?		
	Did you lose your temper or get annoyed over trivial matters?		
	Did you have nightmares or bad dreams?		
	Did you sometimes see or hear things that others could not see or hear?		
	Did trivial things frighten you?		
	Did you sometimes fail to sleep or lose sleep?		
	Were there moments when you felt life was so tough that you cried or wanted to cry?		
	Did you feel run down (tired)?		
	Did you at times feel like committing suicide?		
	Were you generally unhappy with things you were doing each day?		
	Was your work lagging behind?		
	Did you feel you had problems in deciding what to do?		
	Was your stomach/abdomen aching?		

37. Over the last two weeks, how often have you been bothered by any of the following problems?	Not at all (1)	Several days (2)	More than half the days (3)	Nearly everyday (4)
Little interest or pleasure in doing things?				
Feeling down, depressed, or hopeless?				
Trouble falling or staying asleep, or sleeping too much?				
Feeling tired or having little energy?				
Poor appetite or overeating?				
Feeling bad about yourself - or that you are a failure or have let yourself or your family down?				
Trouble concentrating on things, such as reading the newspaper or watching television?				
Moving or speaking so slowly that other people could have noticed?				
Or the opposite - being so fidgety or restless that you have been moving around a lot more than usual?				
Thoughts that you would be better off dead, or of hurting yourself in some way?				
Depression Severity: 0-4 none, 5-9 mild, 10-14 moderate, 15-19 moderately severe, 20-27 severe.				
